# Designing a Band for Vehicles' Drivers Induced by Ultraviolet and Infrared Radiations

**DOI:** 10.1155/2022/7238905

**Published:** 2022-12-21

**Authors:** Nouf Jubran AlQahtani, Amnah Nabil Bukair, Ghada Naje Alessa, Hoor Fayez AlDushaishi, Syed Mehmood Ali

**Affiliations:** Imam Abdulrahman bin Faisal University, Biomedical Engineering Department, P.O. Box: 1982, Dammam 31441, Saudi Arabia

## Abstract

**Background:**

Solar radiations that reach the Earth can be divided into ultraviolet, visible light, and infrared. Overexposure to these radiations can facilitate adverse skin diseases such as sunburn, skin cancer, and photoaging. People who drive vehicles for an extended period are likely to develop skin cancer in the exposed body area.

**Method:**

This research proposes a wearable protective device around the upper arm to measure the transmitted radiation through the front and the side windows. A novel skin type classification algorithm using a color sensor was created to provide an accurate skin type identification. Also, the device was programmed to calculate the time before sunburn occurrence based on the ultraviolet index, sunscreen's sun protection factor, and skin type.

**Results:**

The prototype was tested inside a Toyota Camry model 2001 vehicle with an accuracy of 97%. The front window had transmitted more infrared radiation compared to the side window. The highest recorded value was 76.76 mW/cm^2^. On the other hand, the side window had transmitted more ultraviolet compared to the front window as it lacks the protective polyvinyl butyral layer that the front window has. The highest recorded ultraviolet index was 3.5.

**Conclusion:**

These results highlight the importance of wearing the designed solar band and using appropriate UV and IR protection while driving a vehicle to prevent skin diseases from occurring.

## 1. Introduction

The skin is the largest organ in the body since it accounts for 16% of the body mass. It plays a vital role as an effective physical barrier against the adverse effects of solar radiation. The outermost layer of the skin is the epidermis, mainly comprised of keratinocytes and melanocytes. The keratinocytes provide a physicochemical barrier, and melanocytes produce melanin that absorbs ultraviolet radiation. The inner layer of human skin is the dermis, which primarily constitutes collagen and elastin fibers providing tensile strength and skin elasticity [1, 2].

The solar radiation emitted by the sun includes ultraviolet (UV), visible light (not shown in this work), and infrared (IR) radiation. [3] UV radiations have a shorter wavelength and higher energy absorbed in the skin epidermis by melanin. Melanocytes that release melanin act differently from one person to another. People with darker skin have more melanin pigment production, thus more protection against UV, compared to those with lighter skin. [2] On the other hand, IR radiations have a longer wavelength and lesser energy that allows them to penetrate deeper, reaching the dermis layer rather than being absorbed by the melanin in the basal layer of the epidermis. [4] Previous studies reported a way to protect skin from the harmful effects of UV radiation by applying sunscreen with a high sun protection factor (SPF), which will extend the time before sunburn occurrence. However, its influence is temporary since it lasts for two hours. So, UV radiation has been considered an environmental hazard as excessive exposure to UV radiation appears instantly as a sunburn, whereas repeated exposure can lead to skin cancer [5, 6]. The commercially available sunscreens are not efficient enough to block IR radiations. IR radiations can increase the skin temperature to 43°C and convert it into heat energy. Repeated exposure to IR can promote a skin lesion called erythema ab igne, whereas heat energy cause damage to the dermal structural proteins leading to premature skin aging. [7–9] Overall, the cumulative effects of UV and IR are more detrimental to the skin compared to their influence individually. [10].

Most researchers focus on the direct exposure of solar radiation to the skin. But this work attempts to protect drivers' skin from adverse effects of solar radiation exposure, especially when their profession requires them to spend a significant amount of time inside the vehicles, such as truck and taxi drivers. Generally, solar radiation is transmitted inside the vehicles and contacts the drivers' skin through the front and side window. The front window comprises laminated glass made of two glass layers filled with a polyvinyl butyral (PVB) that absorbs most UV radiation, whereas the side and back windows are composed of tempered glass that lacks a PVB layer and transmits more UV radiation. [11]. Boxer Wachler reported that the side window blockage efficiency is 25% less than the front window [12]. Underestimation of damage induced by IR exposure, even though it has equally harmful effects as UV radiation, may explain the absence of glass that blocks the IR radiation. Although reflective metal IR coatings are available to block IR radiation, they are expensive and rarely used globally. [13] During driving, the driver's part of the skin beside the window is repeatedly exposed to UV and IR radiation in a high dosage, unlike the other side of the body. It is also evident from several studies performed in left-driving countries that the chance of skin cancer increased in the upper left arm, which is one of the most sun-exposed body parts while driving. Moreover, a study in Australia, which is a right-driving country, confirmed that the percentage of skin cancer increased on one side (right side). [14–16].

There is a growing need to develop a low-cost monitoring device for vehicle drivers against the detrimental effects of UV and IR radiation. This research presents a solar band as an alternative solution to minimize the damaging effects of UV and IR radiation on the human skin. Only a few technologies exist that can detect high UV radiations that harm the skin and offer an alert system to seek protection against them. Microsoft band-2 is one of the devices that can measure UV radiations and has an audible alarm. In this band, instead of a time calculation algorithm based on skin types, the user himself defines the reminder period without any consideration of sunburn possibility. [17] UV/IR wristwatch is another device that allows users to enter their skin type with the help of six printed colors in the instruction paper. The algorithm calculates the safe exposure time, and an alarm is activated when exposure time exceeds the defined limits. [18] However, visual anticipation of skin type can carry fault classification and inaccurate safe time calculation, which may confront the user to an exceeded exposure without their knowledge. An alternative solution is to automate the process. The proposed solar band detects the skin color automatically, using the color sensor (TCS34725), and classifies it using a classification algorithm based on cross-sectional data. Nevertheless, none of the commercial devices offer IR monitoring. This device has incorporated the IR and temperature sensor to determine whether IR radiations are high enough to increase skin temperature and damage the skin. The novelty of the solar band is the unprecedented ability to detect radiations from two sides simultaneously. It is designed to be worn around the arm and consists of two sensing panels with a calibrated and tested UV sensor and IR phototransistor. One panel faces the front, and the other faces the side window of the vehicles. The intensities of UV radiations used in the solar band are reported on the UV index scale (UVI), as defined by WHO, ranging from one (low exposure) to 16 (extremely high exposure), and sunburn is associated with UVI. [19] This band calculates the time before sunburn occurrence based on the current UVI, user's skin type, and sunscreen's SPF in case applied. Then, it will activate the alarm when the time before sunburn is exceeded. The solar band is built from an Arduino Nano microcontroller, two UV sensors, two IR phototransistors, a temperature sensor, a color sensor, three pushbuttons to control the device, Nokia 5110 LCD to display data and warning messages, two LEDs, and a buzzer to release an audible alarm. All components are assembled within a case to mimic the function of the solar band.

## 2. Methodology

The solar band measures exposure to UV and IR radiations through car windows and follows two specific algorithms. The first algorithm is the skin color classification that detects the user's skin color and plays a vital role in predicting the danger level. The second algorithm is the time calculation algorithm that calculates the exposure time before sunburn occurs. The components used in this work were chosen after considering several factors, including resource availability, high quality, and low cost. Furthermore, during the developmental process of the prototype, sixty volunteers of different skin types participated in an experiment to collect data for encoding the skin color classification algorithm. Inclusion criteria in the experiment were the agreement for cooperation to participate, healthy skin, and skin color. The volunteers were divided into six equal groups with each skin type (Type 1–Type 6). The experiment was comprehensively explained to them, and signed informed consents were received.

One of the main steps of this work would be designing the solar band or its prototype. The design should consider the components involved and their interaction with each other. Since the solar band needs compact components, which need time to manufacture, a 3D case of the solar band was built of plastic materials using laser cutting machine and components were placed inside it to mimic the function of the solar band. The prototype includes two UV and IR sensors that measure radiation from the front and side windows simultaneously. A temperature sensor was also placed to detect any rise in skin temperature due to IR radiations, along with the color sensor for skin type classification. Nokia 5110 LCD is used as a user interface with a menu list for user interaction. The menu list contains three primary elements. The first element is “Set Skin Type” to detect skin type, while the second is “Set SPF” to select the value of applied sunscreen. The last element is “See Data,” which contains the solar band measurements of UVI, IR, and skin temperature. Finally, this work is executed by assembling all the components with an Arduino microcontroller inside the 3D case. The solar band highlights the damaging effects of solar radiation and protects drivers' skin.

The final step of this work would be testing the prototype and its performance. The test experiment was conducted in Dammam, Saudi Arabia, from 6 : 00 AM to 3 : 00 PM during sunny days of June 2021. The climatic condition of Dammam is desert climate with an average temperature of 26.40°C annually. Then, the performance was assessed inside a Toyota Camry model 2001 vehicle, and the accuracy evaluation was executed using the Solar Light's Model PMA2100 data logger device with two sensors that measure UV and IR radiations. The measurements were taken from both the solar band and data logger simultaneously fixed on the vehicle's front and side window for sixteen minutes, positioned directly to the sun.

### 2.1. Sensors and Measurements

#### 2.1.1. Data Logger

The Solar Light's Model PMA2100 Dual-Input Data Logging Radiometer with two sensors, PMA2140 and PMA2107, was used as a reference device to evaluate the accuracy of the solar band and provide readings in a unit of irradiance (mW/cm^2^). The PMA2140 sensor was used to measure IR radiations and calibrate the BPy62-2 phototransistor. The PMA2107 sensor was used to measure the UV radiations and calibrate the GUVA-S12SD sensor to establish a novel relationship between UVI (UV index) and UV irradiance.

#### 2.1.2. UV Measurements

The GUVA-S12SD sensor was used in the solar band to measure UV radiation in terms of UVI and cover both UVA and UVB spectrum ranging from 240 nm to 370 nm. The photodiode will generate a slight current in response to light irradiance, and the embedded op-amp will magnify the signal in the form of voltage. This sensor produces an analog output signal corresponding to UVI found by multiplying the voltage by 10. If the produced voltage was 0.6 V, the equivalent UVI is six.

The accuracy of the GUVA-S12SD sensor was evaluated by comparing its reading with the reference PMA2140 sensor's reading in terms of irradiance, which will correspond with UVI. The GUVA-S12SD and the radiometer measurements were acquired for five days by placing them under the sun from 6 : 00 AM to 3 : 00 PM during March. Finally, the obtained data were plotted using MS Excel.

#### 2.1.3. IR Measurements

Phototransistor BPy62-2 was used in the solar band to detect the IR spectrum ranging between 700 nm and 1100 nm. The accuracy of the phototransistor was evaluated by comparing it with the reference PMA2107 sensor reading in terms of irradiance, which will be able to correspond to the irradiance with IR voltage. However, the required calibration between the IR irradiance and IR voltage can be achieved only by overcoming the phototransistor saturation problem, so there was a need to attenuate the IR radiations before they reached the sensor. Thus, the aluminum foil was placed over the phototransistor to attenuate some of the IR radiation. Aluminum foil is a conductive material with free electrons in the outer layer, which will vibrate when solar radiation strikes the aluminum. As a result, it will radiate the energy as a reflected wave. [20] The bright side of the aluminum foil is capable of reflecting 57% of near-infrared light. [20] Finally, IR voltage and radiometer measurements were collected simultaneously for five days by placing them under the sun from 6 : 00 AM to 3 : 00 PM during March. Finally, the obtained data were plotted using MS Excel.

#### 2.1.4. Skin Type Classification

Human skin color ranges from dark (categorized as type six) to light skin color (classified as type one), while different color shades represent the other types ranging between them. The skin classification measurements were collected using a TCS34725 RGB color sensor that converts color light to digital output. This sensor is designed for sensing three fundamental colors: red, green, and blue (RGB) via an I2C interface. Every color is a combination of RGB integers ranging from 0 to 255 for each color written as (R, G, *B*), which is the sensor's output data. Ideally, the white color has an RGB code of (255, 255, 255), and the RGB code contains the maximum value of red, green, and blue. In contrast, the black color has an RGB code of (0, 0, 0), where the RGB code includes the minimum values. Thus, the known RGB codes for each skin type that the sensor should read and identify are shown in Table 1.

There were three fundamental stages to calibrate the color sensor and establish accurate readings. The first stage consists of detecting simple objects' color, the second stage includes measurement calibration, and the third stage is validating sensor readings. In the first stage, the color of simple objects, such as colored pens, was measured using the color sensor. The results indicated unrealistic RGB values since the correct RGB value for the green pin should have a higher value of G and almost zeros in R and *B*. The same situation occurred with other pens with different colors, where the sensor output provided inaccurate measurements. So, in the second stage, the color sensor was calibrated to improve the detection capability. The calibration process involves determining the maximum and minimum RGB values by detecting the color of black and white papers. Based on these values, the sensor output is scaled between 0 and -255 by programming simple scaling equations in the Arduino code. The lowest RGB value that the sensor can read is (52, 98, 92), and the highest RGB value is (255, 255, 255). Finally, in the third stage, after calibration, the color sensor was used to test different color pens for validation. As expected, more realistic measurements of the RGB colors were obtained, showing the correct RGB values of the red, green, and blue pens. The results in each stage are illustrated in Table 2.

Once the color sensor is calibrated, the skin measurements are collected for classification purposes. Since each skin color has a different combination of RGB, the RGB codes will differ accordingly. Hence, the sensor should read and identify the RGB code for each skin type. First, the sensor was calibrated before taking the measurements, which is a significant step to ensure high accuracy, standardization, and reliability. Then, skin colors were printed on paper along with their RGB values to ensure the skin RGB colors were measured correctly by the sensor. The difference between RGB values of the printed sample skin colors and observed values from the sensor occurred because the sensor is too sensitive as it relies on the light intensity of the measured color.  Table 3 presents the difference between the exact RGB and the RGB sensor output.

**Figure 1:**
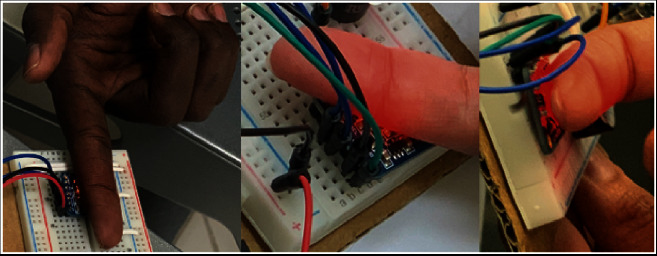
Measuring different skin types using the color sensor, where these measurements were used to build the database that used to classify skin types.

Besides, the object's position to the sensor LED is a significant factor that affects the RGB reading. If the object is too close to the LED sensor, the readings will be more accurate. Although the sensor could not obtain exact RGB measurements of human skin, it offered a remarkable trend in RGB for each skin type based on only the red (R) value in the RGB code. Afterward, a database was created for each skin type to identify the R-value range of the RGB. The database was created by collecting data from the volunteer group of each skin type and analyzing the sensor measurements, as shown in Figure 1.

**Figure 2:**
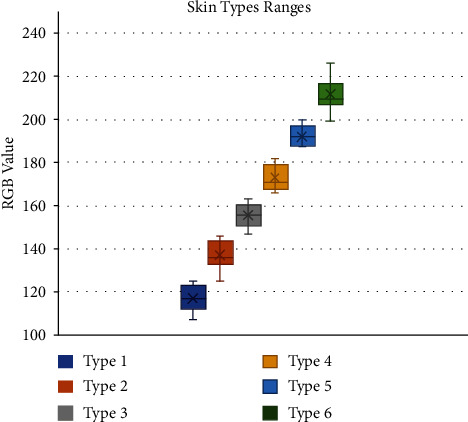
Final ranges of each skin type depending on the database that built using collected measurements from the color sensor.

To build a reliable database that contains all skin tones, ten volunteers participated from each skin type making a total of 60 volunteers. Every volunteer was asked to place the back of their finger on the sensor light to ensure better skin color representation. Fifteen sensor readings were collected from each volunteer to establish an accurate range and decrease the error percentage. Most measurements were within the range that supports the skin type classification method. So, the range selection of each skin type was performed based on the average taken from each reading of the volunteers' skin type.  Figure 2 shows the range selection for sensor programming to classify the user's skin type.

### 2.2. Electrical Connection

**Figure 3:**
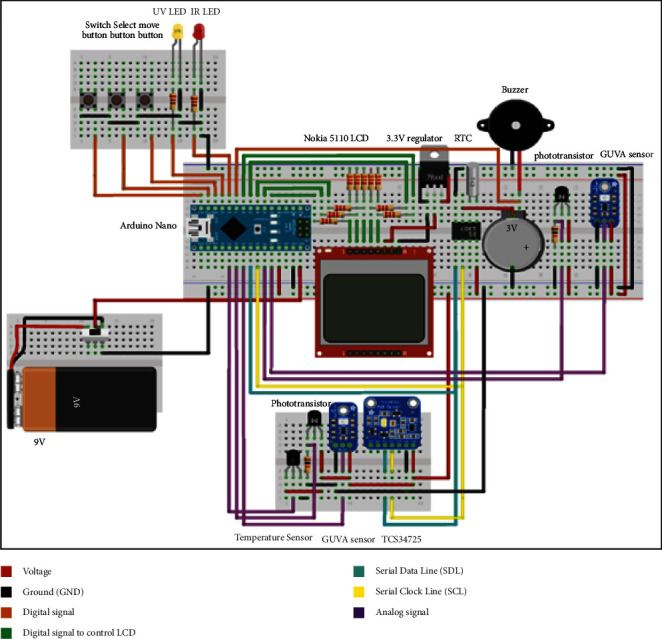
Solar band circuit diagram. The wire's color key is shown on the right side of the diagram.

In this work, Arduino Nano was used to control the circuit. Starting with the power supply, Arduino Nano can be powered with any DC voltage between 6 V and 12 V. The solar band circuit comprises four boards. A switch was placed on the board (B) to turn the ON/OFF solar band, as shown in Figure 3. Moreover, real-time clock (RTC) will need a continuous power supply of 3 V to maintain time calculation even if the circuit is turned off. The clock will be shown on the LCD as an extra feature offered by the solar band. An Arduino Nano, Nokia 5110 LCD, RTC circuit, voltage regulator (LD33 V), Buzzer, IR phototransistor, and GUVA-S12SD sensor were placed on the centered mainboard (*A*). A voltage regulator (LD33 V) was used to supply LCD with a fixed voltage since Nokia 5110 LCD has a maximum voltage of 3.3 V. Besides, a voltage divider circuit was constructed for Arduino logical output connected to Nokia 5110 LCD. Another IR phototransistor and GUVA-S12SD will be placed on board C to face the car's side window when the user wears the solar band around his arm. The color sensor (TCS34725) and temperature sensor (LM35) are placed on the same board. Lastly, the board (*D*) holds three push buttons (Switch, Select, and Move) to control the LCD and two LEDs to indicate the danger existing. These components will be placed on the other side of the board, where the solar radiation is not necessarily reaching these components.

### 2.3. Device Operation

Once the device is operated, LCD will show the “Menu” window and allow user interaction using three pushbuttons. The “Switch” button will allow switching between the “Menu” and “Clock” windows. The “Move” button is used to move between menu options. The “Select” button is used to select an option, access subwindows, and return to the main menu.

**Figure 4:**
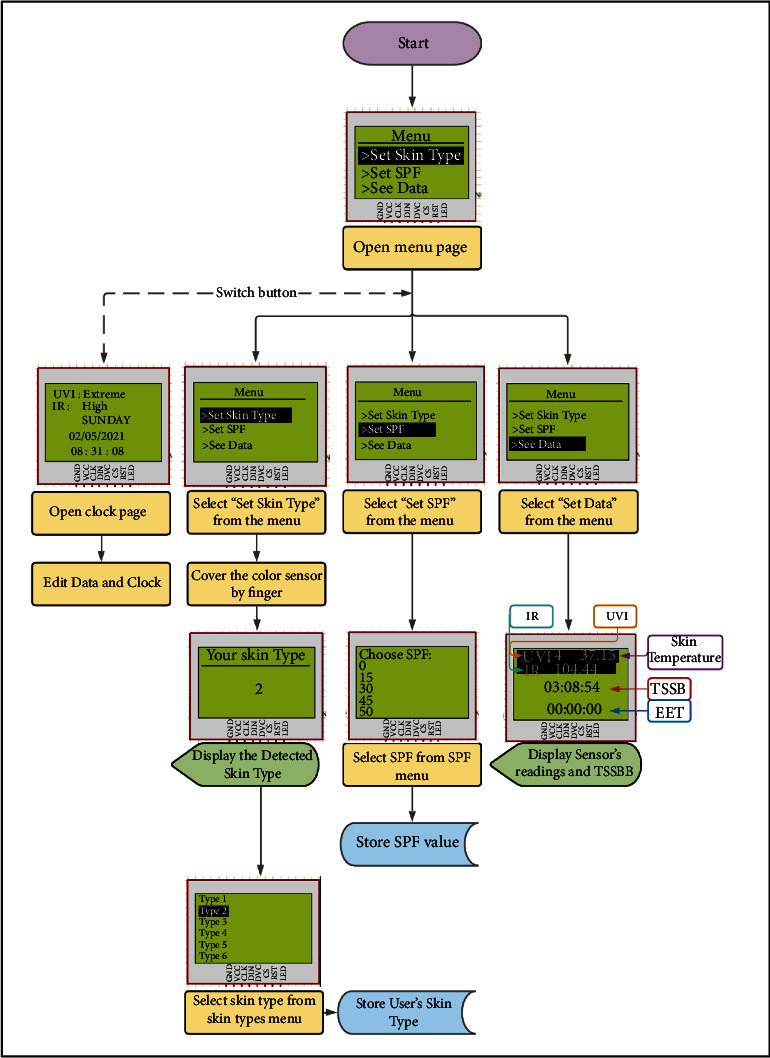
General device operation. Yellow blocks indicate manual operation, pink for input data by sensors, blue for data storage, and green for displaying.

As seen in the flowchart in Figure 4, the “Menu” window will appear once the device is operated. Once the user presses the “Switch” button, the clock window will appear. The user can modify the date and time by pressing the “Select” and “Move” buttons. When the user is satisfied, they can press the “Switch” button to return to the Menu window and enter other windows using the “Select” button.

The menu's first option is titled “Set Skin Type.” After selecting this option, the user will place the dorsal side of their finger over the color sensor. Then, the color sensor will read the skin color, detect the skin type, and display it on the LCD screen. After pressing “Select,” the user will select the skin type from the list, and their skin type will be stored, as shown in Figure 4. The second option is “Set SPF.” It opens a list of SPF values and lets the user selects one value based on the applied sunscreen; then, the program will store the SPF value, as shown in Figure 4. The third option is “See Data.” It shows current UVI, IR, and skin temperature values besides the remaining safe exposure time (TSSB) and exceeded exposure time (EET) in the last row.

#### 2.3.1. Skin Type Classification

**Figure 5:**
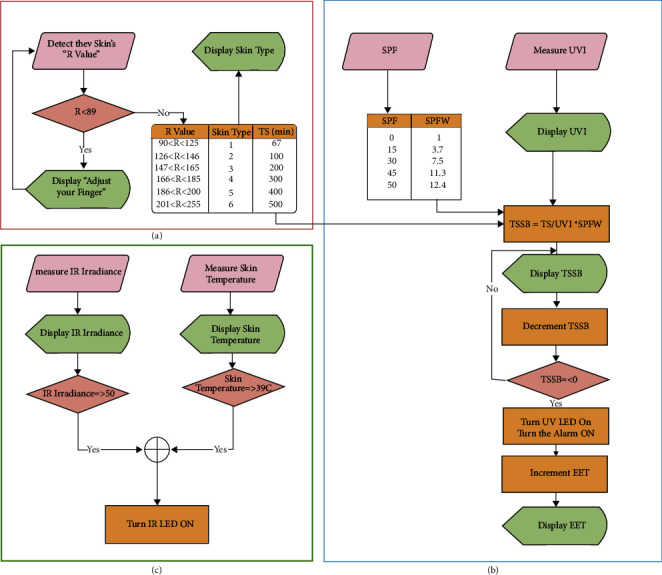
Algorithm behind the solar band. (a) Skin type classification algorithm. (b) Exposure time algorithm. (c) IR alarm algorithm. Pink blocks for input data, red for if conditions, orange for processes, and green for displaying.

The algorithm of the color sensor is summarized in the flowchart, as shown in Figure 5(a). First, the sensor will detect the skin color in terms of RGB. Second, the R-value will be the basis of the skin type classification; it ranges between 0 and 255. R values less than 89 are out of skin type classification ranges. Therefore, the statement “Adjust your finger” will be displayed if the R-value is less than 89. Otherwise, the skin type corresponding to the detected R-value will be displayed, as illustrated by the table in Figure 5(a). For example, if the user has an R-value from 90 to 125, the output will be “Skin Type 1.” Skin type plays a major role in determining the time remaining before sunburn occurrence. For each skin type, a constant called time to sunburn (TS) defines the number of minutes before skin tanning when exposed to UVI of 1. [22, 23] This value will be determined and used to calculate the time before sunburn occurrence (TSSB) for any UVI.

#### 2.3.2. Exposure Time

Sunburn is the immediate reflex of excessive skin exposure to UV, and it indicates that the melanin is no longer protecting the skin. To protect the user from sunburn, the solar band will read UVI, the user's skin type, and the value of the used SPF. After skin type classification, TS will be determined for the user's skin type to calculate the remaining time before sunburn occurs. The time before sunburn occurrence is found by dividing TS by the measured UVI, as illustrated by Equation (1). The six skin types based on Fitzpatrick classification and the time to sunburn for all UVI values are illustrated in Table 4:

(1) $$\begin{array}{c} \text{TSSB }\left(\text{min}\right)=\frac{\text{TS}}{\text{UVI}}\times \text{SPFW.} \end{array}$$

Another factor that affects exposure time is sunscreen. The higher the SPF level, the longer the time to sunburn (TSSB). The users will enter their SPF. Then, the algorithm will define the corresponding sun protection factor weight (SPFW), as shown in Figure 5(b). The corresponding SPFW will be multiplied by time if the user applies sunscreen. Thus, it extends the time before a sunburn, as illustrated by Equation (3). For instance, if a user with skin type two was exposed to UVI of 10 without sunscreen protection, exposure time before sunburn occurrence will be

(2) $$\begin{array}{c} \frac{100\left(\text{min}\right)}{10\left(\text{UVI}\right)}=10\min. \end{array}$$

However, if he applied sunscreen with an SPF of 30, the time to sunburn will be increased by a factor of SPFW and become 75 minutes (1 hour and 15 minutes):

(3) $$\begin{array}{c} \frac{100\left(\text{min}\right)}{10\left(\text{UVI}\right)}\times 7\text{.}5\left(\text{SPFW}\right)=75\text{min.} \end{array}$$

Time to sunburn can extend from 6 minutes to 8 hours, as illustrated in Table 4. During this period, UVI can change, and the user may apply sunscreen with different SPF, which will affect TSSB calculation. Accordingly, an exposure time updating algorithm is implemented to provide the user with the correct information at any time. TSSB will be decremented and displayed every second. Once TSSB is expended, a buzzer will ring, and UV LED will be turned on. After this alarm, the signs of a sunburn may appear as evidence of skin damage due to high UV absorbent. In the long term, repeated excessive exposure may increase the possibility of skin cancer. The critical time after the alarm issuing will be counted as exceeded exposure time (EET) and displayed in the last row in the “See Data” window. The exposure time algorithm is shown in Figure 5(b).

#### 2.3.3. Infrared Alarm

**Figure 6:**
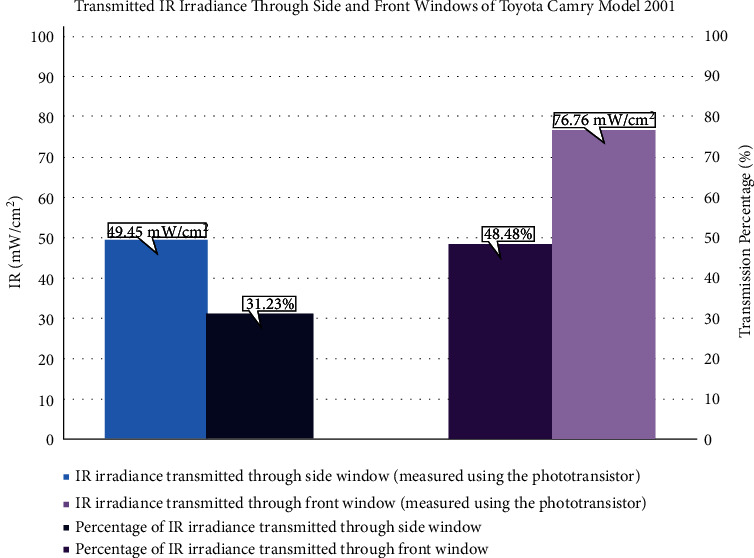
Transmitted IR irradiance through the side (represented in orange color) and front (represented in yellow color) windows in a Toyota Camry model 2001 vehicle.

Infrared has a longer wavelength and deeper penetration through skin layers. They can easily pass through the basal layer, where the melanin pigment exists. As a result, the damage by IR is unaffected by the skin color. The IR phototransistor was calibrated to measure and return IR irradiance in mW/cm^2^ units. High IR irradiance indicates that the IR radiations absorbed by the user's skin cause temperature elevation. While the allowed exposure time was defined based on UVI, there is no clear conclusion on what amount of IR exposure can cause irregular skin temperature increment. This work uses double indication to decide if IR danger exists. First, if the measured IR irradiance reached the threshold defined as 50.42 mW/cm^2^. This value was set based on the average of recorded transmission measurements through the vehicle's front window, as shown in Figure 6. Second, if skin temperature increases beyond 39°C, IR can propagate through the skin, raising the temperature to 40–43°C, where this temperature is associated with irreversible tissue damage. If either condition is true, IR LED will be turned ON, indicating IR threat and the necessity to look for a cold shaded place. The infrared alarm algorithm is shown in Figure 5(c).

#### 2.3.4. Sunscreen Timer

**Figure 7:**
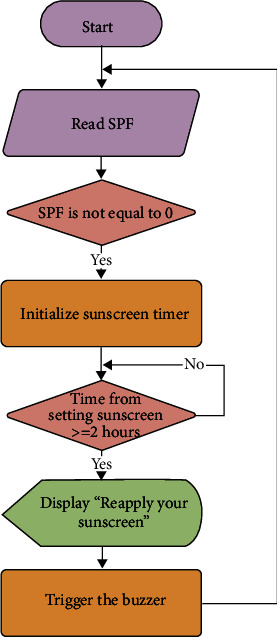
Flowchart of designed algorithm to remind user to reapply sunscreen.

Despite the role of sunscreen in extending the time before sunburn occurrence, its effectiveness lasts two hours after application. Thus, an independent counter will check if two hours is over since the user had entered their SPF to remind the user to reapply for the sunscreen. As shown in Figure 7, the sunscreen timer will be initialized if SPF is not zero. If the time from applying the sunscreen equals or exceeds two hours, the alarm will ring with a statement on the LCD to notify the user. After that, the SPF list will appear so users can enter SPF if they reapplied the sunscreen. If not, SPF will be defined as zero, and the sunscreen timer will not be initialized, but TSSB will be recalculated using the new SPF.

## 3. Results and Discussion

### 3.1. Correlation Analysis

**Figure 8:**
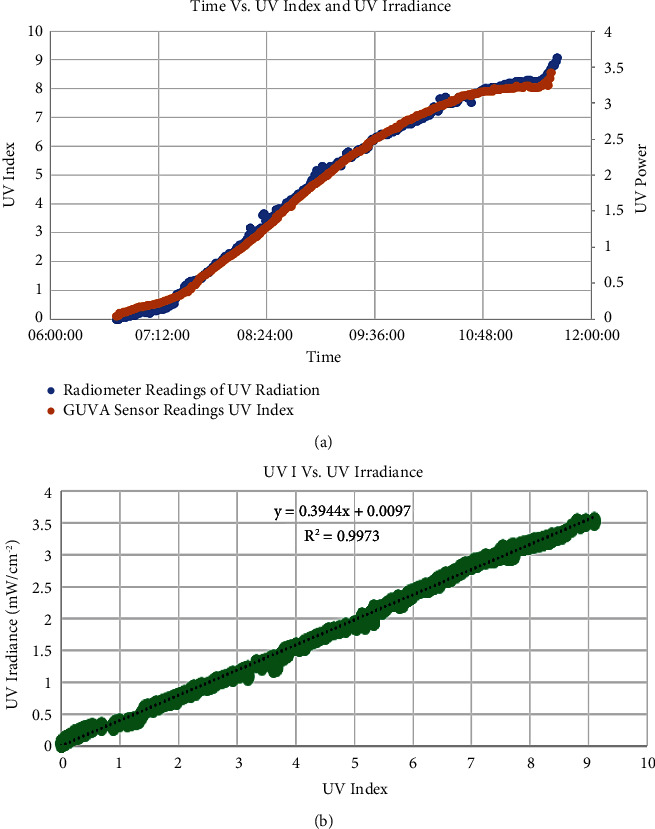
(a) The relationship between time, UV irradiance (blue), and UV index (orange) (b) The linear curve between UVI and UV irradiance.

**Figure 9:**
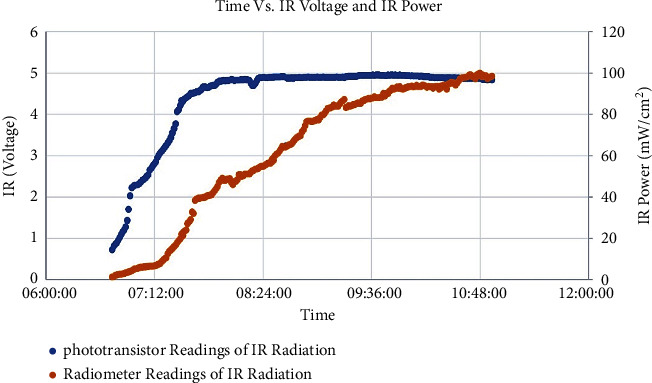
The relationship between time, IR voltage (blue), and IR irradiance (orange).

The correlation between the UV radiation measured by the radiometer and UVI measurements from the GUVA-S12SD sensor is shown in Figure 8(a). Findings indicated a positive linear relationship between them according to the correlation factor value, which is expressed in Equation (4) and shown in Figure 8(b):

In the beginning, establishing the linear relationship between the IR voltage and IR irradiance measured by the radiometer was difficult, as shown in Figure 9. This difficulty is attributed to the saturation of the phototransistor at an IR voltage of 4.55 V, while the corresponding IR irradiance was approximately 42 mW/cm^2^.

**Figure 10:**
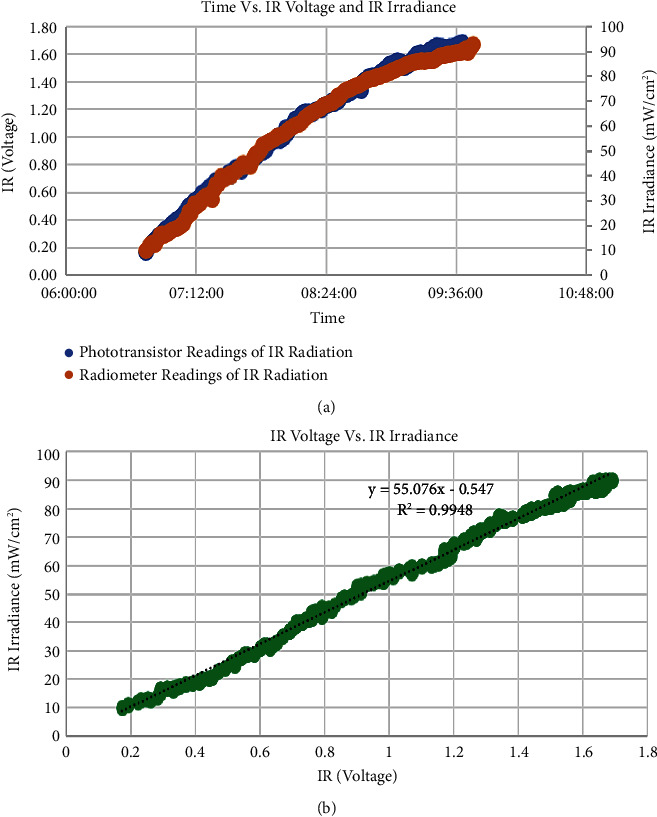
(a) The relationship between time, aluminum-covered phototransistor's voltage (blue), and IR irradiance (orange) (b) The linear curve between IR phototransistor voltage and IR irradiance.

Yet, placing the aluminum foil over the phototransistor helped to find the needed correlation since it played a significant role in preventing the saturation problem. Thus, the linear relationship between phototransistor and radiometer measurements was found, as shown in Figure 10. As a result, the readings will be calibrated even beyond the 42 mW/cm^2^. The correlation factor of the observed relationship was 0.9948, which is expressed in Equation (5) and shown in Figure 10(b):

(4) $$\begin{array}{c} \text{UV Irradiance}\left(\frac{\text{mW}}{{\text{cm}}^{2}}\right)=\text{0.}3944\times \text{UVI}+\text{0.}0097\text{,} \end{array}$$

(5) $$\begin{array}{c} \text{IR irradiance}\left(\frac{\text{mW}}{{\text{cm}}^{2}}\right)=55\text{.}076\;\times \text{voltage}+\text{0.}547\text{.} \end{array}$$

#### 3.1.1. Prototype Design

**Figure 11:**
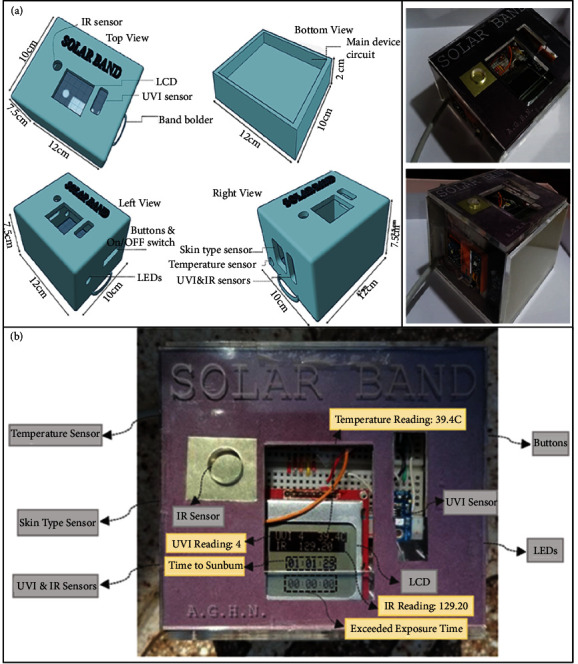
(a) 3D design of prototype with upper and lower views with real images. (b) The solar band prototype with labeled components (grey boxes) and UVI, IR irradiance, and temperature reading (yellow boxes).

One of the main goals of this work was to design a prototype that combines all the solar band components. An important design consideration is a small size to be worn on the user's arm or wrist. However, the designed case is larger than it should be for containing all the electric components. The upper cover has three openings from the top for the LCD, UV sensor, and IR sensor. Also, it has three openings from the right side for the skin type sensor, temperature sensor, UV, and IR sensors. While from the left, it has two holes: one for the buttons and the ON/OFF switch, and the other for the LEDs. The designed prototype case from the top, bottom, right, and left views is shown in Figure 11(a). The solar band prototype is shown in Figure 11(b).

#### 3.1.2. Prototype Operation

**Figure 12:**
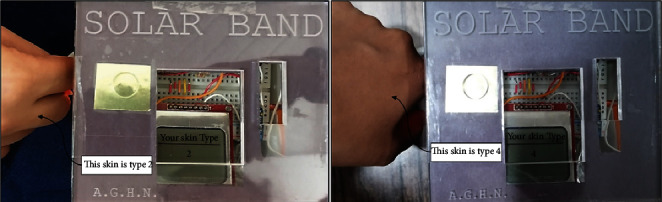
Detected skin type of volunteers using the prototype.

The UVI and IR radiation measurements were recorded using the solar band, as shown in Figure 11. The IR irradiance was 129.20 mW/cm^2^, whereas the skin temperature reached 39.4°C. The UVI, IR, temperature, time to sunburn, and exceeded exposure time are displayed on the LCD screen. Automatic skin type classification is the critical and unprecedented feature in the proposed solar band. The skin types two and four of the volunteers were detected according to skin type classification, as shown in Figure 12.

#### 3.1.3. Prototype Experiments

It has been evidenced from the studies that the drivers are not protected from UV and IR radiation. These radiations can penetrate car windows, increasing the chance of skin damage and even skin cancer. [24] As we cannot escape the UV and IR rays from the sun, this research has led to a significant development that can help protect people from these radiations.

**Figure 13:**
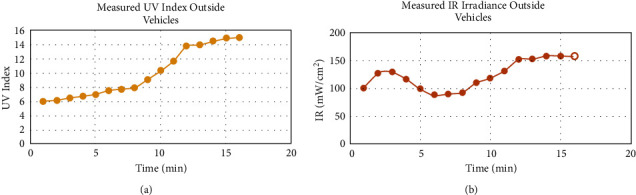
(a) UVI measurements outside the vehicles for sixteen minutes. (b) IR measurements outside the vehicles for sixteen minute.

A series of experiments were performed during the summer from 9 : 00 AM to 3 : 00 PM in Dammam, Saudi Arabia, to evaluate the adverse effects of solar radiation. Since one of the distinctive features of the designed device is the ability to measure UV and IR radiations inside vehicles, the prototype was tested inside a Toyota Camry model 2001 vehicle. The vehicle was directed to the sun. The UV and IR measurements were recorded outside and inside the car for comparison purposes. The data logger device was used for the reference measurements of UV and IR radiations. The measurements were taken for 16 minutes, where the value of UVI increased as the time passed. The maximum outside UVI recorded was 14.9, while the lowest was 6.02, as shown in Figure 13. The IR measurements recorded during the 16 minutes showed fluctuation between the maximum reading of 158.33 mW/cm^2^, and the minimum of 89.24 mW/cm^2^, as shown in Figure 13.

**Figure 14:**
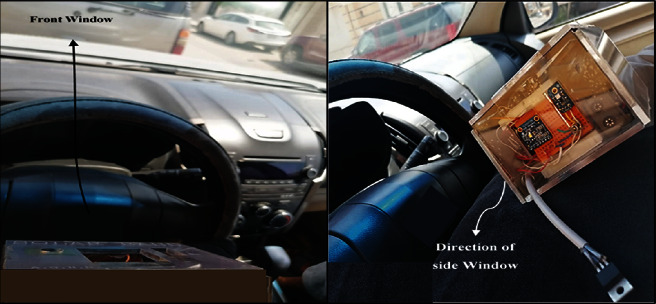
Orientation of prototype inside the vehicle, the top view oriented to the front window, while the left side view oriented to the side window.

Afterward, the solar band was employed inside the vehicle to measure transmitted UV and IR radiations through the vehicle's windows, as shown in Figure 14.

**Figure 15:**
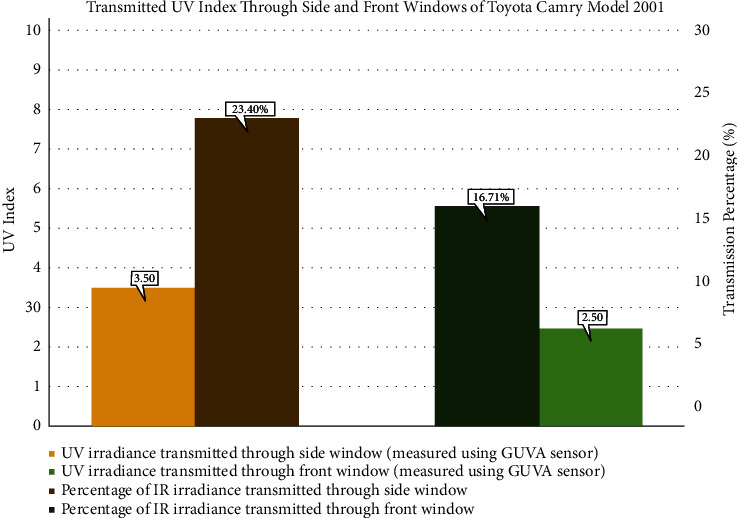
Transmitted UV index through the side (represented in green color) and front (represented in blue color) windows in a Toyota Camry model 2001 vehicle.

The vehicle transmitted a high UVI of 2.5 value through the front window, which was attributed to the less efficient PVB layer. Furthermore, the UVI value transmitted through side windows was higher than the front window since the front window is treated to shield drivers from some UV radiations, unlike side windows. It can be observed from the experiments that the obtained results were in agreement with previous findings, which state that the side window can transmit UV radiation more than the front window because it lacks the PVB layer. The measurements of transmitted UVI through Toyota Camry model 2001 windows are shown in Figure 15. Moreover, the figure represents the transmission percentages through side and front windows compared to outside UVI.

The IR transmission measurements through the side and front windows were 49.45 mW/cm^2^ and 76.76 mW/cm^2^, respectively, as shown in Figure 6. The observed results indicate a high amount of IR that can reach the driver in this vehicle, specifically through the front window. As mentioned before, the front window of the cars does not include any specific material or layer that blocks or reflects the IR radiation. Therefore, a high amount of IR radiation is transmitted via the front window.

This study is the first conducted in Saudi Arabia that measures UVI and IR radiation transmission through vehicles and their impacts on the driver's skin. The previous research was in Mexico [25] and showed that the front window blocks UV more than the side window, which agrees with this study. However, the average transmission percentage of UV in the Mexico study was 16%, while it reached 23.40% in this study. The results suggest that excessive UV and IR radiations transmitted significantly through the side window harm the driver's skin leading to sunburn. But, the accumulative exposure may result in other skin pathologies, such as early aging and skin cancer. The time duration will vary according to the skin type, where types one, two, and three are more susceptible to having early sunburn within approximately 17 to 67 minutes. Skin types four, five, and six may take more time to develop sunburn within 75 to 167 minutes, as shown in Table 5. This variation happened due to melanin level; as melanin production level is high, sunburn is unlikely to happen. These results verified that the time to a sunburn could be reached within a day, indicating that the person with skin types one, two, and three can be affected by repeated sunburn that leads to severe consequences, such as skin cancer. As declared before, the highest recorded value of IR outside the vehicles is equal to 158.33 mW/cm^2^, and the Toyota Camry model 2001 vehicle transmits 48.48% of the radiation through the front window and 31.23% through the side windows, which indicates that excessive exposure can lead to skin disorders. This experiment proved that even with the PVB layer existing in the front window, the UV radiation substantially transmits from the side window and reaches the driver. Moreover, the PVB layer does not provide a barrier to IR radiation. So, a high amount of IR radiation is transmitted via front and side windows. Although people are usually aware of the devastating effects of UV radiation, they underestimate the IR radiation implications on human skin.

#### 3.1.4. Prototype Accuracy

Reliability and efficiency are significant factors to consider during the designing and testing process of any device. Therefore, the data logger device was used as a reference device to test the prototype. This process was performed by taking measurements of UV and IR radiations in the Toyota Camry model 2001 vehicle using both devices (data logger and prototype). After the measurements were taken, the UV power was calculated using Equation (5) and then compared with the recorded one by the data logger device. The measurements showed that the percentage error was small since it did not exceed 5%.

A comparison between the prototype and the reference device was performed by calculating the difference between the two measurements. This variation might be attributed to uncontrolled factors, such as the human factor, efficiency of used sensors in prototypes, and experiments setup. In general, the efficiency of sensors decreases with time and frequent use; therefore, errors may occur. Also, the data logger sensors have a wider diameter, which means they can cover enough areas to detect correct measurements with sun movements. Moreover, the vehicle's orientation could affect the results. For example, if the car's direction faces the sun, more radiation would reach the driver. Nevertheless, the percentage error of the prototype was acceptable since its efficiency was estimated as 97%.

## 4. Conclusion

Even though humans constantly need sunlight, some harmful UV and IR radiations reach the Earth since the ozone layer cannot block them. Thus, cumulative exposure to harmful UV and IR radiations leads to dangerous complications, especially in the skin, such as sunburn, photoaging, and skin cancer. This work presents an electrical device that measures UV and IR radiations, calculates the allowed exposure time, and sends an alarm when the time is exceeded. A color sensor was used to detect the user's skin type by an algorithm to identify skin type. The sensors used for detecting UV and IR radiations were well-calibrated. The prototype was tested inside a vehicle, where the results show the importance of the proposed device since IR and UV can transmit through the vehicle windows. Finally, the accuracy was tested, and the maximum percentage error of the proposed device compared to the reference device was 3.37%, which indicates that the efficiency of the proposed device is not less than 97%. The future work includes designing a band with microelectronic components and expanding car experiments to cover many vehicles with different models. Also, a phone app will be designed to transfer and store exposure data to allow dermatology revision. It can be helpful for people with a cancer history or immune deficiency.

## Figures and Tables

**Table 1:** RGB values of the Fitzpatrick scale [21].

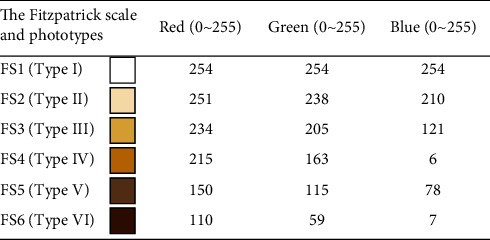

**Table 2:** The three main stages of color sensor measurements and their corresponding output.

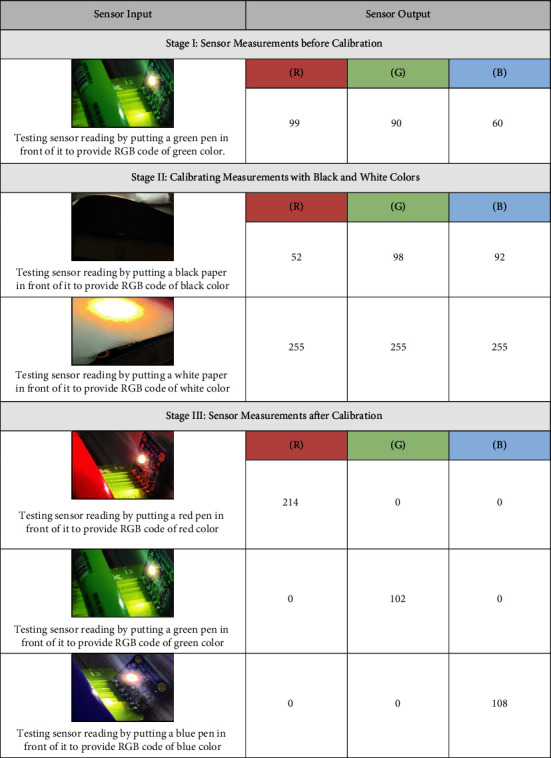

**Table 3:** The difference between the exact RGB codes of skin color and the sensor reading for similar colors.

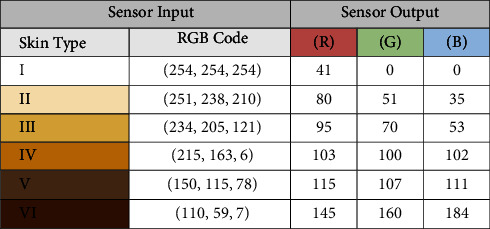

**Table 4:** Time to sunburn in minutes for six skin types without sunscreen at different UVI [22, 23].

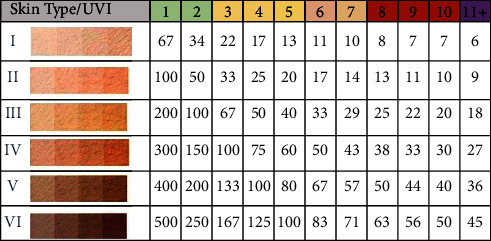

**Table 5:** Transmitted UVI through a Toyota Camry model 2001 vehicle, and the corresponding time to sunburn for skin types 1 to 6.

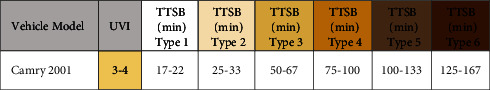

## Data Availability

The data used to support the findings of this study are available from the corresponding author upon request.
